# A Broad Spectrum Racemase in *Pseudomonas putida* KT2440 Plays a Key Role in Amino Acid Catabolism

**DOI:** 10.3389/fmicb.2018.01343

**Published:** 2018-06-29

**Authors:** Atanas D. Radkov, Luke A. Moe

**Affiliations:** Department of Plant and Soil Sciences, College of Agriculture, University of Kentucky, Lexington, KY, United States

**Keywords:** D-amino acids, Pseudomonas, racemase, metabolism, peptidoglycan

## Abstract

The broad-spectrum amino acid racemase (Alr) of *Pseudomonas putida* KT2440 preferentially interconverts the l- and d-stereoisomers of Lys and Arg. Despite conservation of broad-spectrum racemases among bacteria, little is known regarding their physiological role. Here we explore potential functional roles for Alr in *P. putida* KT2440. We demonstrate through cellular fractionation that Alr enzymatic activity is found in the periplasm, consistent with its putative periplasm targeting sequence. Specific activity of Alr is highest during exponential growth, and this activity corresponds with an increased accumulation of d-Lys in the growth medium. An *alr* gene knockout strain (Δ*alr*) was generated and used to assess potential roles for the *alr* gene in peptidoglycan structure, producing soluble signaling compounds, and amino acid metabolism. The stationary phase peptidoglycan structure did not differ between wild-type and Δ*alr* strains, indicating that products resulting from Alr activity are not incorporated into peptidoglycan under these conditions. RNA-seq was used to assess differences in the transcriptome between the wild-type and Δ*alr* strains. Genes undergoing differential expression were limited to those involved in amino acid metabolism. The Δ*alr* strain exhibited a limited capacity for catabolism of l-Lys and l-Arg as the sole source of carbon and nitrogen. This is consistent with a predicted role for Alr in catabolism of l-Lys by virtue of its ability to convert l-Lys to d-Lys, which is further catabolized through the l-pipecolate pathway. The metabolic profiles here also implicate Alr in catabolism of l-Arg, although the pathway by which d-Arg is further catabolized is not clear at this time. Overall, data presented here describe the primary role of Alr as important for basic amino acid metabolism.

## Introduction

d-Amino acids (d-AAs) are typically less abundant than their L-amino acid (l-AA) counterparts, but they are increasingly seen as important effectors of bacterial physiology (Lam et al., 2009; Leiman et al., 2013). The key structural roles of d-Ala and d-Glu in peptidoglycan are well appreciated, and additional d-AAs (e.g., d-Lys, d-Ser, and d-Met) have been identified in peptidoglycan from diverse bacteria (Vollmer et al., 2008). In addition to their roles in bacteria, d-AAs are now known to play important roles in higher organisms, including humans (Genchi, 2017). Recent work has shed light on the expanding roles of d-AAs in modulating bacterial behavior, including biofilm dispersal and growth phase transition (Lam et al., 2009; Leiman et al., 2013). Further, d-AAs are found in abundance in microbe-rich environments, including soils and fermented foods (Brückner and Hausch, 1989; Vranova et al., 2012), where they are presumed to originate from microbial activity. Nonetheless, with the exception of d-Ala and d-Glu, little is known concerning the mechanisms and reasons for d-AA synthesis (Radkov and Moe, 2014b).

Pseudomonads are good models for studying the biology of d-AAs (Li and Lu, 2018), as they are proficient catabolizers and synthesizers of d-AAs and metabolic pathways have already been worked out for several (Revelles et al., 2007; Li and Lu, 2009; Watanabe et al., 2012; He et al., 2014; Nikel et al., 2015; Belda et al., 2016; Chavarría et al., 2016). Previous work from our lab established that the *alr* gene from *Pseudomonas putida* KT2440 encodes a broad-spectrum AA racemase (BSAR) that exhibits the highest activity interconverting the l- and d-stereoisomers of Lys (Radkov and Moe, 2013). Because *P. putida* KT2440 catabolizes d- and l-Lys using independent mechanisms, it has been suggested that the Alr enzyme may be responsible for linking the two pathways (Revelles et al., 2007). Indeed, catabolism of l-AAs in some cases is initiated by conversion to the corresponding d-AA [e.g., l-Ala catabolism in *Salmonella typhimurium, Escherichia coli, Pseudomonas aeruginosa* PAO1, and *P. putida* KT2440 (Wasserman et al., 1983; Lobocka et al., 1994; He et al., 2011)].

In addition to its proposed role in Lys catabolism, additional roles may be considered for Alr and other BSARs. These potential functions include metabolic (e.g., synthesis/catabolism of other AAs), structural (e.g., synthesis of d-AAs for peptidoglycan), or signaling roles (e.g., production of d-AAs as soluble signals).

Here we explore potential functions in signaling, peptidoglycan structure, and AA metabolism in *P. putida* KT2440 using a markerless *alr* deletion in conjunction with biochemical and transcriptomic analysis. Periplasmic activity of the Alr enzyme is demonstrated, but the enzyme has no apparent impact on peptidoglycan structure under the conditions tested. RNA-seq data does not show evidence that the products of the Alr enzyme induce expression of any genes beyond those necessary for catabolism of Lys, pointing to a primarily metabolic role for the Alr enzyme in *P. putida* KT2440.

## Methods

### Construction of markerless mutation via short overlap extension PCR (SOE PCR)

Primers down_cut (CTAGGAATTCTAATGCGCTCGGCGACGTCA, EcoRI cut site is underlined) plus down_homology (**ATGCCCTTTCGCCGTACC**AAGATACTCGTCGACTGA, bold type—start of *alr* gene, regular type—end of *alr* gene) and up_cut (CGTCAAGCTTCTTCATCAGCAGCGACATG, HindIII cut site is underlined) plus up_homology (TCAGTCGACGAGTATCTT**GGTACGGCGAAAGGGCAT**, regular type—end of *alr* gene, bold type—start of *alr* gene) were used in the initial round of PCR to provide fragments downstream and upstream of the *alr* gene, and also engineer an overlap sequence (primers down_homology and up_homology are the reverse complement of each other and contain 36 nucleotides of the *alr* gene). Overnight culture of *P. putida* KT2440 supplied the template DNA (2 μl). Following the initial PCR, the SOE PCR was performed using ~20 ng of gel purified DNA (Thermo Scientific GeneJET gel extraction kit) from each of the two above reactions, also using primers down_cut and up_cut. All reactions were completed using PrimeSTAR HS DNA Polymerase (TaKaRa) according to the provided instructions (50°C annealing temperature). The SOE PCR provided a fragment containing 500 bp upstream and downstream of *alr*, including an engineered restriction enzyme cut site on each end, as well as 18 nucleotides at the beginning and end of the *alr* gene. Parts of the *alr* gene (36 nucleotides total) were not deleted in order to achieve an in-frame deletion and also minimize any impact on the transcription of upstream and downstream genes. Vector pEX18Kan [(Choi and Schweizer, 2005), kindly provided by Virginia Stockwell, Oregon State University] and the gel purified SOE PCR fragments were digested using the appropriate restriction enzymes (Thermo Scientific FastDigest). The digested products were purified (Thermo Scientific PCR purification kit) and subsequently ligated using T4 DNA ligase. The ligation reaction was kept for 2 h at room temperature and overnight at 4°C. The ligation reactions were desalted and heat inactivated (10 min at 65°C) prior to electroporation into *E. coli* cells (Epicentre; TransforMax EPI300 Electrocompetent *E. coli*). Positive clones were selected on media containing kanamycin (30 μg ml^−1^). The pEX18Δ*alr* construct was confirmed using colony PCR (Thermo Scientific; DreamTaq Green DNA PCR Master Mix) and DNA sequencing (Applied Biosystems; BigDye Terminator, v3.1, cycle sequencing kit) with the M13-27 and M13-20 primers (Integrated DNA Technologies). Subsequently, the purified construct (Thermo Scientific GeneJET plasmid miniprep kit) was electroporated into *P. putida* KT2440 electrocompetent cells prepared according to Choi and Schweizer (Choi and Schweizer, 2005). Between 5 and 10 μl of purified plasmid were added (300–500 ng) and the cells were electroporated (Bio-Rad GenePulser Xcell; Voltage-1,800 V, Capacitance-25 μF, Resistance-200 Ω, 1 mm cuvette), followed by an immediate addition of 1 ml LB and 1 h recovery in a shaker at 28°C. The entire recovery mixture was plated on a single LB plus kanamycin plate (30 μg ml^−1^) and incubated at 28°C. To distinguish between single- and double-crossover events, 1 ml LB aliquots, without any selection, were inoculated with individual transformants and incubated at 28°C with shaking for 2 to 3 h. After the incubation time, 100 μl of a 10-fold dilution were plated onto LB plates containing 5% (w/v) sucrose. Several colonies were restreaked onto LB/Kan (30 μg ml^−1^) and LB + 5% (w/v) sucrose to confirm their inability to grow in the presence of Kan selection, suggesting that they no longer carry the Kan resistance cassette. Several colonies were selected as potential deletion mutants. The markerless deletion was confirmed via colony PCR and sequencing of the amplified DNA The seq_down, GATCATGGGCAACGCACAG and seq_up, CATCCGCAATGGGCATG primers were used, with expected fragment sizes of 1.2 kb for the deletion mutant and 2.4 kb for wild-type (wt) revertants.

### Periplasmic protein isolation and enzyme assays

Periplasmic protein was isolated according to the chloroform extraction method (Ames et al., 1984; Klotz and Hutcheson, 1992). Briefly, a 5 ml culture (stationary growth phase, OD_600_ > 1.0, and exponential growth phase, OD_600_ = 0.6) was harvested via centrifugation. The pelleted cells were washed twice using buffer A2X [50 mM HEPES pH 7.4, 200 mM NaCl, 1.95 mM Tris (2-carboxyethyl) phosphine hydrochloride (TCEP), 10% glycerol (v/v)]. After completing the washes, the cells were resuspended in 800 μl cold buffer A2X and 30 μl chloroform was added. The cell mixture was vortexed briefly and maintained on ice for 15 min. The treated cells were centrifuged at 10,600 × g (Eppendorf Centrifuge 5424) at 4°C for 10 min. Five-Hundred microliter of the supernatant was transferred to a new tube and kept on ice. This fraction represents “periplasmic” protein. The remaining, intact cell pellet was washed twice with 1 ml buffer A2X and then resuspended in 1 ml of the same buffer. The cells were lysed by sonication twice at 60 V for 10 s with a 2 min pause on ice in between. The lysate was centrifuged at 4°C at maximum speed (15,700 × g; Eppendorf Centrifuge 5424) and 500 μl of the supernatant was transferred to a new tube and maintained on ice. This fraction represents the “cytoplasmic” protein, which includes intracellular protein as well as any remaining periplasmic protein. The identity of each protein fraction, periplasmic and cytoplasmic, was confirmed via enzyme assays using glucose-6-phosphate dehydrogenase (cytoplasmic localization; Klotz and Hutcheson, 1992) and alkaline phosphatase (periplasmic localization; Junker and Ramos, 1999). The assays were performed at 37°C for 1 h in a total volume of 200 μl. The substrates used were *para*-nitrophenyl phosphate (Sigma) for the alkaline phosphatase enzyme and glucose-6-phosphate/NADP^+^ (Sigma) for glucose-6-phosphate dehydrogenase (both substrates were in 50 mM HEPES buffer, pH 7.4). The enzyme assays were quenched with 40 μl 1 M Na_2_CO_3_. *P. putida* wt as well as Δ*alr* cultures at exponential and stationary growth phases were used to isolate periplasmic and cytoplasmic protein, which was subsequently used to determine Alr racemase activity according to Radkov and Moe (Radkov and Moe, 2013, 2014a).

### Peptidogycan isolation and composition analysis

Our method was adapted from Boniface et al. (2009) where it was used to demonstrate the presence of d-Lys as part of the peptide stem of *Thermotoga maritima* peptidoglycan. Three 50 ml LB cultures per strain (24 h of shaking at 220 rpm at 28°C-stationary phase at OD_600_ > 1.0) were harvested in 50 ml tubes and resuspended in 5 ml 20 mM KH_2_PO_4_ (pH 7.2) with 1 mM DTT. The cells were sonicated (ten cycles at 60 V for 20 s with 2 min pause on ice in between each cycle) and Triton X-100 was added to 0.5% final concentration (v/v). The suspension was vortexed for 30 s and incubated at 55°C for 30 min. The treated lysate was centrifuged at 200,000 × g at 20°C for 20 min (Beckman Coulter Optima L-100 XP; Type 70Ti rotor). The recovered pellet was rinsed four times with 1 ml water without resuspension and then centrifuged once more using the same parameters. The pellet was resuspended in 1 ml 0.1 M ammonium acetate, pH 6.0. The pellet was broken using the pipette tip during resuspension, as the material becomes highly compacted after centrifugation. The resuspended pellet was incubated with 1 mg ml^−1^ lysozyme (Research Product International) at 37°C with shaking (220 rpm) for 2.5 h. After incubation, the mixture was centrifuged at 18,000 × g for 10 min and transferred to a new tube. Two volumes of cold acetone were added, the mixture was vortexed for 30 s and then centrifuged at 18,000 × g. The entire supernatant was transferred to a glass vial and evaporated overnight under air. The remaining solid was resuspended in 900 μl 6M HCl, transferred to a polypropylene 2 ml microfuge tube and incubated at 95°C in a hot plate for 12 to 16 h. After hydrolysis, the solution was cooled to room temperature and added to 900 μl 6M NaOH. The resulting solution was derivatized using Marfey's reagent (Sigma) and analyzed via HPLC (Radkov and Moe, 2013, 2014a). The elution time of each detected peak was compared to an authentic AA standard derivatized and analyzed in the same manner as the sample.

### *In vivo* Lys racemization

Wt and Δ*alr P. putida* KT2440 5 ml cultures were started using LB medium and OD_600_ was recorded (Biotek Synergy HT plate reader) after 2, 4, 6, 8, and 10 h of incubation at 28°C with shaking. The cells in each culture were pelleted via centrifugation and the supernatant was derivatized using Marfey's reagent and analyzed via HPLC as described above [see (Radkov and Moe, 2014a) for a detailed protocol].

### Gene expression analysis

*P. putida* KT2440 wt and Δ*alr* 5 ml LB cultures were started from an isolated colony on an LB agar plate. After incubating for 24 h, 100-fold dilution was performed to prepare fresh 1 ml LB cultures of each strain. Three biological replicates were included for each strain. After incubating for 6 h at 28°C with shaking, RNA was isolated from each culture using the RNeasy mini kit (Qiagen) according to the manufacturer's instructions. cDNA libraries were prepared using an established protocol (Hunt, 2015). Briefly, 2,000 ng RNA was treated with DNase I (Invitrogen) in order to remove any residual genomic DNA. The samples were then treated with the rRNA RiboMinus kit (Invitrogen) according to the manufacturer's instructions to remove 16S and 23S rRNA and subsequently purified using an RNeasy mini kit. The RNA was analyzed on a 2% agarose gel before and after each of the above treatments to observe the major rRNA bands. SMARTSCRIBE Reverse Transcriptase (Clontech) was used for cDNA synthesis with Superase∙ In (LifeTechnologies) used as RNase inhibitor. The following primers were used for the construction of each library: Reverse Transcription primer was 5′-ACACTCTTTCCCTACACGACGCTCTTCCGATCTXXXNNNNNN-3′ (where XXX symbolizes the sample barcodes-wt1-CCA, wt2-GTG, wt3-AAT; Δ*alr*1-TGC, Δ*alr* 2-CGG, Δ*alr*3-GAA, and NNNNNN indicating random hexamer). The template-switching primer was CGGTCTCGGCATTCCTGCTGAACCGCTCTTCCGATCTGG+G (the last position is occupied by a locked nucleic acid). Following cDNA synthesis, the samples were purified using AMPure magnetic beads (Beckman Coulter). Lastly, the cDNA was enriched using Phire Taq polymerase (Thermo-Fischer) and the following Illumina-specific PCR primers 5′-AATGATACGGCGACCACCGAGATCTACACTCTTTCCCTACACGACGCTCTTCCGATCT-3′ and 5′-CAAGCAGAAGACGGCATACGAGATCGGTCTCGGCATTCCTGCTGAACCGCTCTTCCGATCT-3′. Each library was assessed on a 2% agarose gel and the number of PCR cycles was varied until a smear was obtained in the 200–500 bp range according to agarose gel results. AMPure magnetic beads (Beckman Coulter) were used to purify the enriched DNA samples. DNA quantification, library pooling, and MiSeq sequencing were performed at the Advanced Genetics Technologies Center at University of Kentucky. Gene expression analysis was conducted using the CLC Genomics Workbench software (CLC bio). RNA-seq data has been submitted to the NCBI BioProject database under accession PRJNA471301.

### Growth assays

A collection of 19 d-AAs and their corresponding l-stereoisomers (19 chiral proteinogenic AAs) were used to determine the growth profile for *P. putida* KT2440 and Δ*alr* strains (Figures S1, S2). Each AA was tested as the sole source of C and N. Sterile PG minimal medium was employed as the base medium (Studier, 2005). The final AA concentration was 25 mM except for Tyr, used at 2.5 mM due to limited solubility. This concentration was chosen to ensure robust growth. The assays were performed in 96-well microplates (CytoOne; Flat bottom, polystyrene) in 200 μl volume maintained at 28°C with shaking (220 rpm). For each AA, three replicates were prepared and an uninoculated well was included as a growth control. Each replicate was inoculated using 1 μl of an overnight culture in LB washed twice with sterile PG containing no N or C source. Absorbance measurements at 600 nm were performed using a plate reader (Biotek Synergy HT) after 24, 48, and 72 h. The uninoculated wells for each AA were used as a blank. After collecting the necessary data, direct PCR using seq_down and seq_up primers (see above) verified that each culture contained the appropriate strain (Δ*alr* or wt *P. putida* KT2440) according to the PCR band size.

## Results

### Alr enzyme activity

Based on previous work (Muramatsu et al., 2005), we hypothesized that the Alr enzyme would be localized to the periplasmic space in *P. putida* KT2440. We used the chloroform method for obtaining the protein fraction, referred to here as “periplasmic,” that is released upon incubation of the cells with chloroform. The cellular fraction that remained was lysed to obtain the “total” fraction that comprised remaining periplasmic protein as well as the cytoplasmic protein. The goal of this experiment was to determine whether Alr activity could be detected in the periplasmic fraction, and therefore to confirm previous suppositions about its localization. Because of the nature of the fractionation, it is not possible to completely exclude cytoplasmic protein from the “periplasmic” fraction, nor is it possible to exclude periplasmic protein from the “total” fraction. Therefore, we sought to determine the relative quantities of cytoplasmic protein in the “periplasmic” fraction and the relative quantities of periplasmic protein in the “total” fraction using assays to detect the relative activity of a known cytoplasmic enzyme (glucose-6-phosphate dehydrogenase, G6PDH) in both “periplasmic” and “total” fractions and the relative activity of a known periplasmic enzyme (alkaline phosphatase, AP) in the “periplasmic” and “total” fractions. It was not our goal, using this method, to exclude Alr activity in the cytoplasm, only to detect its activity in the periplasm. Based on the relative activity of AP in the “periplasmic” and “total” fractions (Table S1), it is clear that the “periplasmic” fraction did indeed contain protein known to be localized to the periplasm (AP), and that the “total” fraction retains significant periplasmic protein. With AP activity normalized to 100% in the “total” fraction, the “periplasmic” fraction had 72–77% of the AP activity. Based on the relative activity of G6PDH in the “periplasmic” and “total” fractions, it is clear that some cytoplasmic protein is present in the “periplasmic” fraction, albeit at a much lower level than in the “total” fraction. The key point from this assay is that the G6PDH activity in the “periplasmic” fraction is relatively minor (13.3–16.7%) compared to its activity in the “total” fraction (normalized to 100%). As such, we consider the relative amounts of cytoplasmic protein in the “periplasmic” fraction to be minor, and the relative amounts of periplasmic protein in the “total” fraction to be fairly high.

With this in mind, Alr activity was highly abundant in the “periplasmic” fraction of *P. putida* KT2440 during stationary phase (specific activity of 134.5 ± 29.8 nmol min^−1^ mg^−1^) compared to the activity in the “total” fraction (209.7 ± 8.2 nmol min^−1^ mg^−1^). Normalizing the “total” fraction to 100% activity, this gives the “periplasmic” fraction approximately 64% activity, which is in line with the relative activity of AP in the “total” vs. “periplasmic” fraction stated above (72–77%). No Alr activity was detected in either the “periplasmic” or “total” fractions from the Δ*alr* mutant strain.

### *In vivo* Lys racemization and peptidoglycan structure

**Figure 1:**
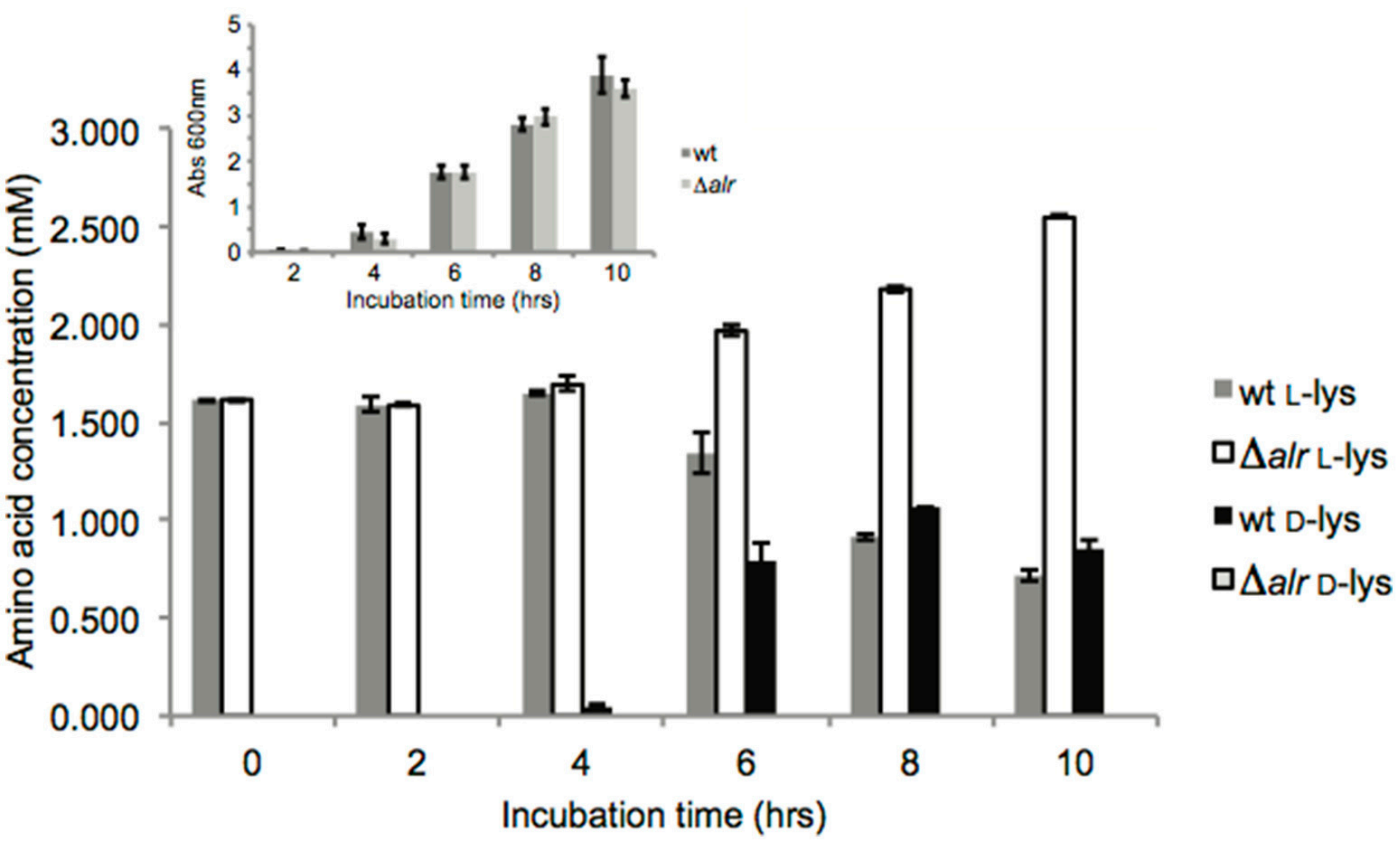
Lys racemization *in vivo*. Average concentration values ± SD are the result of three biological replicates with three technical replicates each. Inset: growth measurements (OD_600_) were performed on the same cultures. No statistically significant (*p* > 0.05) OD_600_ differences were found.

Alr activity was monitored *in vivo* by measuring d- and l-Lys quantities in the growth medium when the wt and Δ*alr* strains were grown in LB medium (Figure 1). No significant differences were observed for overall growth (measured as optical density at 600 nm) between the wt and Δ*alr* strains during the course of the experiment (Figure 1, inset). A small amount of d-Lys (less than 100 μM) was detected in the wt growth medium after 4 h of incubation (Figure 1), and the d-Lys concentration increased after that, reaching ~1 mM after 8 h. The corresponding levels of l-Lys decreased as d-Lys increased. In the Δ*alr* strain, no d-Lys was detected during the course of the study, but the levels of l-Lys in the medium increased from ~1.5 mM at *t* = 0 to 2 mM at 8 h and 2.5 mM at 10 h (Figure 1). The combined level of d- and l-Lys in the wt was similar to the level of l-Lys in the Δ*alr* strain up to the 8 h time point. However, this was not the case after 10 h of incubation, as the combined concentration of d-Lys + l-Lys for the wt was 1.5 mM while the concentration of l-Lys for the Δ*alr* strain reached 2.5 mM. This implies that *P. putida* KT2440 secretes l-Lys, even under conditions of high Lys content in the medium, and that the rate of total Lys consumption is greater under conditions when Alr is active (i.e., wt strain).

Due to the presence of d-Lys in the supernatant and the periplasmic localization of the Alr enzyme, we next considered whether Alr activity had an impact on peptidoglycan structure. No significant differences (*p* > 0.05) were observed in the stationary phase composition of peptidoglycan between the wt and Δ*alr* strains (Table 1), precluding a role for Alr in peptidoglycan modification under these conditions. Both strains had approximately equal amounts of the conventional peptidoglycan stem AAs l-Ala, d-Glu, and d-Ala (Vollmer et al., 2008). The predicted crosslinking AA d,l-diaminopimelic acid was also seen, along with small amounts of l-Arg and l-Lys. No d-Lys was detected. Other as-yet-unidentified peaks were observed in the HPLC chromatogram, however none of those showed significant differences (*p* > 0.05) between the two strains.

**Table 1:** AAs detected in peptidoglycan and culture supernatant.

	**Peptidoglycan^a^**	
**AA/Strain**	**wt**	**Δ*alr***
	**Percent of identified AA content^b^**	
l-Ala	24.2 ± 1.6	24.0 ± 2.6
d-Glu	22.9 ± 0.6	23.9 ± 3.2
d, l-Diaminopimelic acid	13.2 ± 0.3	14.0 ± 0.8
d-Ala	22.4 ± 0.0	22.3 ± 0.5
l-Lys	4.0 ± 1.5^c^	7.2 ± 1.6^c^
d-Lys	ND	ND
l-Arg	13.2 ± 0.8	13.1 ± 0.3
d-Arg	ND	ND

a *Measured during stationary phase, 50 ml culture, LB medium, 28°C, 220 rpm*.

b *Calculated as percent of the total area of the AAs shown in the table – the respective total area was the same between the two peptidoglycan fractions, as well as the same between the three supernatant fractions*.

c *This difference in the amount of L-Lys is not statistically significant (p > 0.05, Student's t-test). Average values of three replicates ± standard deviation are shown. ND, AA was not detected*.

### Gene expression analysis between Wt and Δ*alr*

**Figure 2:**
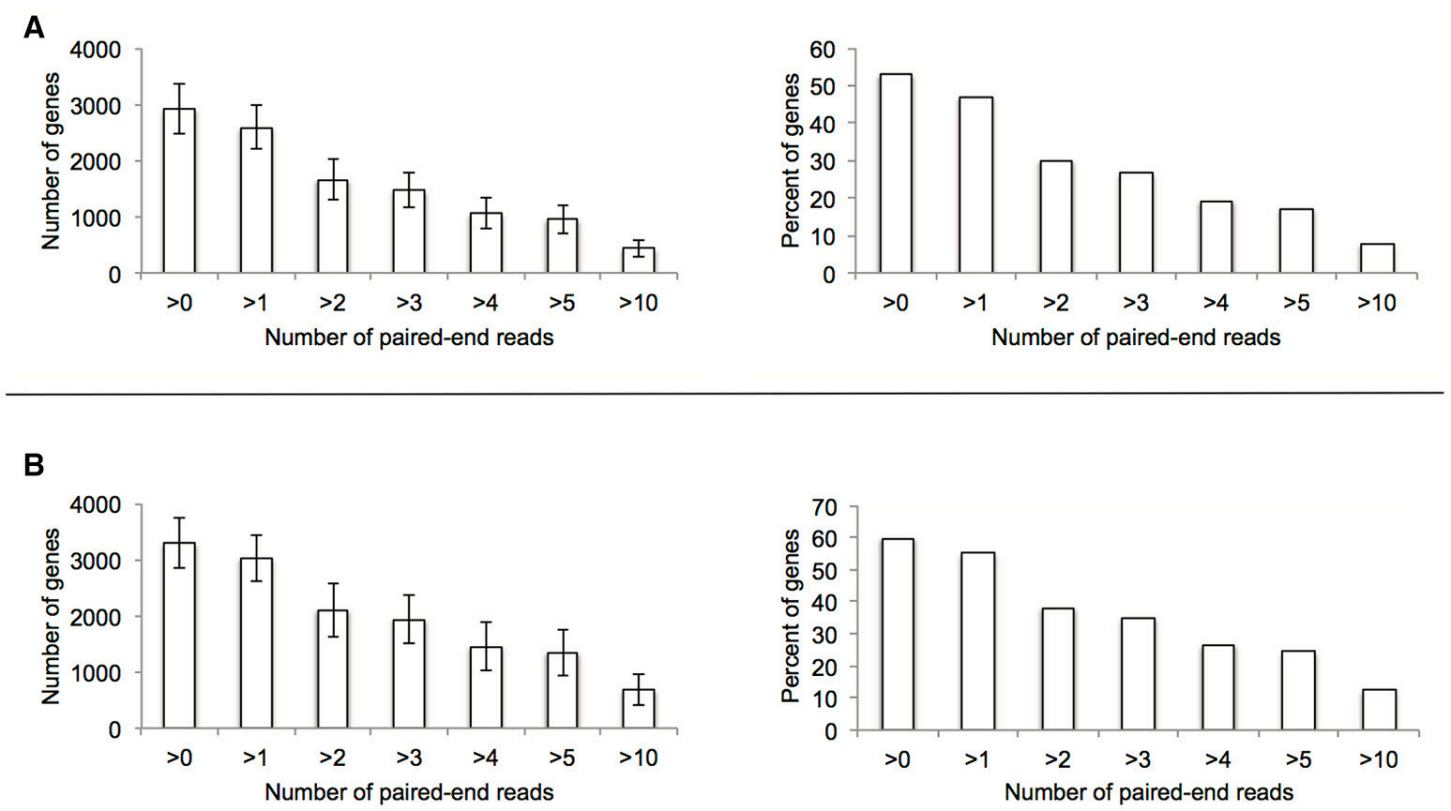
Summary of genome coverage RNA-seq data used for the comparative experiment between wt **(A)** and Δ*alr* **(B)** strains.

**Figure 3:**
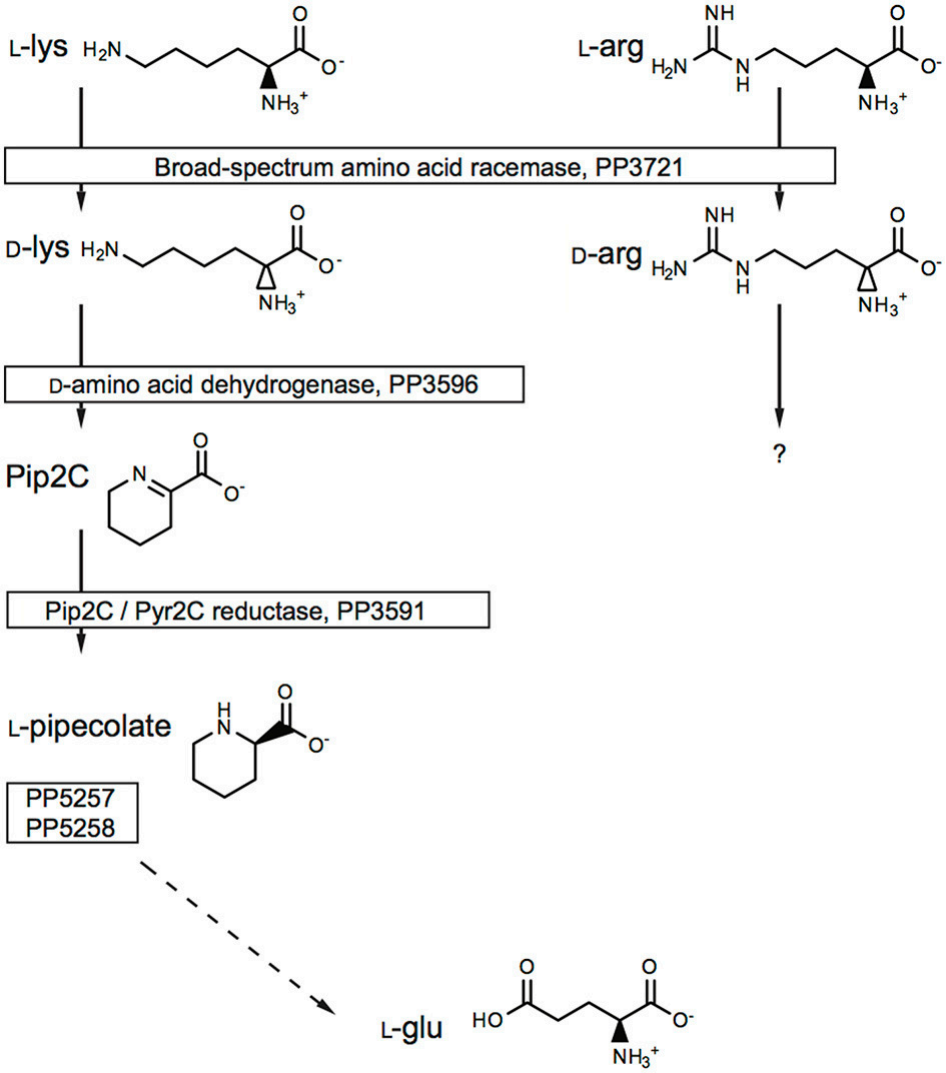
The role of the Alr BSAR in l-Lys and l-Arg catabolism. The gene numbers were obtained from the Pseudomonas Genome Database.

To explore the potential for the Alr enzyme and its products as signals that may induce gene expression, we performed a comprehensive gene expression analysis with the wt and Δ*alr* strains using RNA-seq. More than half of the *P. putida* KT2440 genome was covered by at least one paired-end read and approximately 10% by 10 or more paired-end reads (Figure 2). The genes included in Table 2 (12 genes of 119 total with statistically significant expression, *p* < 0.05) have RPKM values (reads per kilo base per million mapped reads) larger than 200 and fold-change differences higher than 3. Several genes known to be involved in d-Lys catabolism were significantly overexpressed (*p* < 0.05) in the wt relative to the Δ*alr* strain (Table 2). These genes encode the d-AA dehydrogenase which converts d-Lys to Δ^1^-piperideine-2-carboxylate (Pip2C, gene PP3596), the Δ^1^-piperideine-2-carboxylate/Δ^1^-pyrroline-2-carboxylate reductase (Pip2C/Pyr2C reductase), which converts Pip2C into l-pipecolate (PP3591), and downstream genes acting on l-pipecolate and subsequent catabolites (PP5257 and PP5258). These activities can be mapped to the Lys catabolism pathway from Figure 3. The difference in RPKM for genes overexpressed in the wt varies from ~100 (PP3722 and PP3596) to ~1,600 (PP5258). The most highly overexpressed gene in the wt strain compared to the Δ*alr* strain was PP3593 (fold-change 27), which is a putative AA transporter/AA-binding protein localized to the periplasm (Table 2). Although this gene has not been characterized biochemically, it appears to be part of an operon with PP3596, also overexpressed in the wt strain (fold-change 6), which is involved in d-Lys catabolism (Revelles et al., 2007). Only four genes were overexpressed in the Δ*alr* strain relative to the wt (Table 2), and their RPKM and fold-change values were generally lower than the genes overexpressed in the wt strain. The only information pertaining to functions of these genes relates to a homolog of gene PP2656, which is shown to have a role in biofilm formation in *P. aeruginosa* (Duque et al., 2013).

**Table 2:** Comparison of gene expression between the wt and Δalr strain.

**Genes overexpressed in the wt strain compared to Δ*alr***								
**Localization**	**Cluster of orthologous group (COG)**	**Feature ID**	**Function or conserved domain database (CDD) predicted function**	**wt**	**Δ*alr***	**Difference**	**Fold change**	***P*-value**
Periplasmic	Amino acid transport and metabolism; Signal transduction mechanisms	PP_3593	Part of d-Lys catabolism	426.28	15.95	410.33	27	0.02
**Periplasmic**	**Cell wall/membrane/envelope biogenesis**	**PP_3722**	**Part of d-Lys catabolism**	**102.51**	**7.07**	**95.43**	**14**	**0.04**
Cytoplasmic membrane	Energy production and conversion	PP_3591	Part of d-Lys catabolism	304.20	22.82	281.37	13	0.02
Cytoplasmic	Amino acid transport and metabolism	PP_3191	CDD: AA-ammonia lyase	446.67	45.24	401.43	10	0.00
Cytoplasmic	Energy production and conversion	PP_5258	Part of d-Lys catabolism	1795.84	201.07	1,594.77	9	0.02
Cytoplasmic	Function unknown	PP_5260	CDD: domain of unknown function DUF 1338	245.25	30.65	214.60	8	0.01
Cytoplasmic	Amino acid transport and metabolism	PP_5257	Part of d-Lys catabolism	516.49	85.62	430.87	6	0.01
**Cytoplasmic membrane**	**Amino acid transport and metabolism**	**PP_3596**	**Part of d-Lys catabolism**	**114.49**	**18.81**	**95.69**	**6**	**0.05**
**Genes overexpressed in the Δ*alr* strain compared to wt**								
Unknown	Function unknown	PP_3743	CDD: domain of unknown function DUF2388	69.99	334.68	264.69	5	0.01
Cytoplasmic	Inorganic ion transport and metabolism	PP_1082	CDD: bacterioferritin	142.78	397.85	255.07	3	0.02
Cytoplasmic membrane	Cell wall/membrane/envelope biogenesis	PP_1600	CDD: periplasmic chaperone (part of cell wall synthesis operon)	205.13	526.66	321.53	3	0.04
**Periplasmic**	**Inorganic ion transport and metabolism**	**PP_2656**	**CDD: penicillin-binding protein-like**	**371.69**	**879.53**	**507.84**	**2**	**0.04**

*Values for wt and Δalr represent reads per kilo base transcript per million mapped reads (RPKM). Shown are the average values, derived from the gene expression analysis using three unique cDNA libraries for each strain. All of the included genes here have RPKM values larger than 200 and a fold-change of 3 or higher, except for the genes in bold—they have either RPKM values higher than 200 or a fold-change of 3 or higher*.

### Impact of *alr* deletion on AA catabolism

The *alr* deletion did not impact whether a particular AA was used as a C and N source, but the levels of growth differed in some cases. Of the 19 tested d-AAs, six were catabolized as the sole C and N source, while the remaining 15 conferred negligible growth (Table 3). Most of the l-AAs (15 l-AAs) were catabolized as the sole source of C and N, while six l-AAs (Cys, Leu, Met, Thr, Trp, and Tyr) yielded minimal growth (Figures S1, S2). These data are consistent with previous observations on AA catabolism by *P. putida* KT2440 (Radkov and Moe, 2013).

**Table 3:** Growth profile of Pseudomonas putida KT2440 wt and Δ*alr* strains on 19 d- or l-AAs as a sole source of C and N.

	**wt**	**Δ*alr***	**wt**	**Δ*alr***
	**C and N source, d-AAs**		**C and N source, l-AAs**	
Ala	+++	+++	+++	+++
Arg	++	++	+++	+
Asn	–	–	+++	+++
Asp	–	–	+++	+++
Cys	–	–	–	–
Glu	–	–	+++	+++
Gln	–	–	+++	+++
His	–	–	+++	+++
Ile	–	–	++	++
Leu	–	–	–	–
Lys	++	++	++	+
Met	–	–	–	–
Phe	+	+	+	+
Pro	–	–	+++	+++
Ser	–	–	++	++
Thr	–	–	–	–
Trp	–	–	–	–
Tyr	–	–	–	–
Val	–	–	++	++

*+++, abs at 600 nm ≥ 0.5 after 24 h of incubation; ++, abs at 600 nm ≥ 0.5 after 48 h of incubation; +, abs at 600 nm ≥ 0.5 after 72 h of incubation; –, abs at 600 nm < 0.5 after 72 h of incubation; Growth assays were performed in triplicate. The shading highlights the only significant differences (p < 0.05, Student's t-test) between the two strains*.

Differences were noted between the wt and Δ*alr* strains in their levels of growth using l-Arg and l-Lys as the sole source of C and N (Table 3). In each case, the wt strain exhibited significantly greater growth relative to Δ*alr*. The same trends were not observed with the corresponding d-AAs, implying that these AAs are catabolized, at least in part, through their d-AA enantiomer.

## Discussion

BSARs with enzymatic properties similar to Alr have been reported for several organisms, including *Vibrio cholerae, Proteus mirabilis, Oenococcus oeni*, and *Pseudomonas taetrolens* (Matsui et al., 2009; Kuan et al., 2011; Kato et al., 2012; Espaillat et al., 2014). Despite available biochemical work, there is little information on the specific biological role of these enzymes. *P. putida* KT2440 encodes distinct mechanisms for catabolism of l- and d-Lys, and some evidence suggests that the Alr racemase links the two pathways (Revelles et al., 2005, 2007). Other work, however, implicates a BSAR in synthesis of d-AAs (d-Met and d-Leu) in *V. cholera*e (Lam et al., 2009; Espaillat et al., 2014). In the case of *V. cholerae*, the d-AAs alter cell morphology by virtue of their incorporation into peptidoglycan. We therefore sought to explore potential roles for the *P. putida* KT2440 Alr enzyme in AA catabolism as well as d-AA synthesis.

Consistent with its putative periplasmic target sequence, the Alr enzyme was found to be active in periplasmic fractions from both exponential and stationary phase cultures. As expected the Δ*alr* strain showed no Alr activity, and accumulated no d-Lys in the LB growth medium. Appearance of d-Lys in the wt medium coincided with the period of rapid growth, leveling off as the culture entered stationary phase. The appearance of additional Lys in the medium suggests that it is exported from the cell where it is converted to d-Lys, such that the amount of d-Lys exceeds l-Lys as the cell enters stationary phase. Owing to the timing of the d-Lys accumulation, we hypothesized that it may act as a soluble signal to induce gene expression, akin to quorum sensing, or that it may be incorporated into peptidoglycan. We found no evidence to suggest that any products of Alr activity were incorporated into peptidoglycan (Table 1). RNA-seq data from wt and Δ*alr* cells grown under the same conditions as those that produced d-Lys showed that the majority of the genes (with known function) upregulated in the wt strain related to catabolism of AAs, suggesting that under these growth conditions the products of Alr activity do not appear to impact properties beyond metabolism of AAs.

From our observations, elimination of Alr activity had the most significant impact on the metabolic capacity of *P. putida* KT2440. The Δ*alr* strain showed diminished capacity for catabolism of the two l-AAs that are the best substrates for Alr (Arg and Lys), implying that in each case conversion to their corresponding d-enantiomer promotes catabolism. Previous work has shown that Alr is involved in catabolism of l-Lys (Revelles et al., 2005, 2007), but this is the first work to show that this enzyme may also be involved in catabolism of l-Arg. Work by Li and Lu has demonstrated a two-enzyme pathway (DauBAR pathway) for conversion of d-Arg to l-Arg for subsequent catabolism in *Pseudomonas aeruginosa* (Li and Lu, 2009). This pathway is not present, based on comparative genomic analysis, in *P. putida* KT2440 (Radkov and Moe, 2013). To our knowledge, a separate pathway for d-Arg catabolism, independent of l-Arg, has not been characterized. Further work will be required to determine the mechanism of d-Arg catabolism in *P. putida* KT2440.

Data presented here support a role for Alr in the catabolism of l-Lys and l-Arg through conversion to their respective d-enantiomers. While the d-AAs resulting from Alr activity are produced, at least partly, in the periplasm, they are not incorporated into peptidoglycan under these conditions. Further, despite accumulation of d-Lys in LB growth medium, this does not appear to act in any broader signaling capacity. Rather, the excess of d-Lys promotes expression of genes that are necessary for d-Lys catabolism. At this time, however, it is not clear why Alr is localized to the periplasm in *P. putida* KT2440 when the d-AAs produced are further catabolized in the cytoplasm. This work adds to a growing body of data indicating that d-AAs play myriad roles in the physiology of bacteria.

## Author contributions

LM and AR designed the experimental work. AR performed the work.

### Conflict of interest statement

The authors declare that the research was conducted in the absence of any commercial or financial relationships that could be construed as a potential conflict of interest.

## Supplementary material

Figure S1 OD600 values for growth of wt and Δ*alr* strains on 19 L-AAs.

Figure S2 OD600 values for growth of wt and Δ*alr* strains on 19 D-AAs.

Table S1 Protein fraction identification using known enzyme activity localization.

## References

[B1] Ames G. F. Prody C. Kustu S. (1984). Simple, rapid, and quantitative release of periplasmic proteins by chloroform. J. Bacteriol. 160, 1181–1183. 6501229 10.1128/jb.160.3.1181-1183.1984 PMC215841

[B2] Belda E. van Heck R. G. A. José Lopez-Sanchez M. Cruveiller S. Barbe V. Fraser C.. (2016). The revisited genome of *Pseudomonas putida* KT2440 enlightens its value as a robust metabolic chassis. Environ. Microbiol. 18, 3403–3424. 10.1111/1462-2920.13230 26913973

[B3] Boniface A. Parquet C. Arthur M. Mengin-Lecreulx D. Blanot D. (2009). The elucidation of the structure of *Thermotoga maritima* peptidoglycan reveals two novel types of cross-link. J. Biol. Chem. 284, 21856–21862. 10.1074/jbc.M109.034363 19542229 PMC2755910

[B4] Brückner H. Hausch M. (1989). Gas chromatographic detection of d-amino acids as common constituents of fermented foods. Chromatographia 28, 487–492. 10.1007/BF02261066

[B5] Chavarría M. Goñi-Moreno Á. de Lorenzo V. Nikel P. I. (2016). A metabolic widget adjusts the phosphoenolpyruvate-dependent fructose influx in *Pseudomonas putida*. mSystems 1, 1–15. 10.1128/mSystems.00154-16 27933319 PMC5141268

[B6] Choi K.-H. Schweizer H. P. (2005). An improved method for rapid generation of unmarked *Pseudomonas aeruginosa* deletion mutants. BMC Microbiol. 5:30. 10.1186/1471-2180-5-30 15907219 PMC1173109

[B7] Duque E. de la Torre J. Bernal P. Molina-Henares M. A. Alaminos M. Espinosa-Urgel M.. (2013). Identification of reciprocal adhesion genes in pathogenic and non-pathogenic Pseudomonas. Environ. Microbiol. 15, 36–48. 10.1111/j.1462-2920.2012.02732.x 22458445

[B8] Espaillat A. Carrasco-López C. Bernardo-García N. Pietrosemoli N. Otero L. H. Álvarez L.. (2014). Structural basis for the broad specificity of a new family of amino-acid racemases. Acta Crystallogr. 70, 79–90. 10.1107/S1399004713024838 24419381 PMC4984259

[B9] Genchi G. (2017). An overview on d-amino acids. Amino Acids 49, 1521–1533. 10.1007/s00726-017-2459-5 28681245

[B10] He W. Li C. Lu C.-D. (2011). Regulation and characterization of the dadRAX locus for d-amino acid catabolism in *Pseudomonas aeruginosa* PAO1. J. Bacteriol. 193, 2107–2115. 10.1128/JB.00036-11 21378189 PMC3133094

[B11] He W. Li G. Yang C.-K. Lu C.-D. (2014). Functional characterization of the dguRABC locus for d-glutamate and d-glutamine utilization in *Pseudomonas aeruginosa* PAO1. Microbiology 160, 2331–2340. 10.1099/mic.0.081141-0 25082951

[B12] Hunt A. (2015). A rapid, simple, and inexpensive method for the preparation of strand-specific RNA-seq libraries. Methods Mol. Biol. 1255, 195–207. 10.1007/978-1-4939-2175-1_17 25487215

[B13] Junker F. Ramos J. (1999). Involvement of the cis/trans isomerase Cti in solvent resistance of *Pseudomonas putida* DOT-T1E. J. Bacteriol. 181:5693. 10482510 10.1128/jb.181.18.5693-5700.1999 PMC94089

[B14] Kato S. Hemmi H. Yoshimura T. (2012). Lysine racemase from a lactic acid bacterium, *Oenococcus oeni*: structural basis of substrate specificity. J. Biochem. 152, 505–508. 10.1093/jb/mvs120 23035128

[B15] Klotz M. G. Hutcheson S. W. (1992). Multiple periplasmic catalases in phytopathogenic strains of *Pseudomonas syringae*. Appl. Environ. Microbiol. 58, 2468–2473. 1514792 10.1128/aem.58.8.2468-2473.1992 PMC195805

[B16] Kuan Y.-C. Kao C.-H. Chen C.-H. Chen C.-C. Hu H.-Y. Hsu W.-H. (2011). Biochemical characterization of a novel lysine racemase from *Proteus mirabilis* BCRC10725. Proc. Biochem. 46, 1914–1920. 10.1016/j.procbio.2011.06.019

[B17] Lam H. Oh D.-C. Cava F. Takacs C. N. Clardy J. de Pedro M. A.. (2009). d-Amino acids govern stationary phase cell wall remodeling in bacteria. Science 325, 1552–1555. 10.1126/science.1178123 19762646 PMC2759711

[B18] Leiman S. A. May J. M. Lebar M. D. Kahne D. Kolter R. Losick R. (2013). d-Amino acids indirectly inhibit biofilm formation in *Bacillus subtilis* by interfering with protein synthesis. J. Bacteriol. 195, 5391–5395. 10.1128/JB.00975-13 24097941 PMC3837952

[B19] Li C. Lu C.-D. (2009). Arginine racemization by coupled catabolic and anabolic dehydrogenases. Proc. Natl. Acad. Sci. U.S.A. 106, 906–911. 10.1073/pnas.0808269106 19139398 PMC2630070

[B20] Li G. Lu C.-D. (2018). Molecular characterization and regulation of operons for asparagine and aspartate uptake and utilization in *Pseudomonas aeruginosa*. Microbiology 164, 205–216. 10.1099/mic.0.000594 29293081

[B21] Lobocka M. Hennig J. Wild J. Klopotowski T. (1994). Organization and expression of the *Escherichia coli* K-12 dad operon encoding the smaller subunit of d-amino acid dehydrogenase and the catabolic alanine racemase. J. Bacteriol. 176, 1500–1510. 10.1128/jb.176.5.1500-1510.1994 7906689 PMC205218

[B22] Matsui D. Oikawa T. Arakawa N. Osumi S. Lausberg F. Stäbler N.. (2009). A periplasmic, pyridoxal-5′-phosphate-dependent amino acid racemase in *Pseudomonas taetrolens*. Appl. Microbiol. Biotechnol. 83, 1045–1054. 10.1007/s00253-009-1942-7 19300994

[B23] Muramatsu H. Mihara H. Kakutani R. Yasuda M. Ueda M. Kurihara T.. (2005). The putative malate/lactate dehydrogenase from *Pseudomonas putida* is an NADPH-dependent Δ^1^-piperideine-2-carboxylate/Δ^1^-pyrroline-2-carboxylate reductase involved in the catabolism of d-lysine and d-proline. J. Biol. Chem. 280, 5329–5335. 10.1074/jbc.M411918200 15561717

[B24] Nikel P. I. Chavarría M. Fuhrer T. Sauer U. De Lorenzo V. (2015). *Pseudomonas putida* KT2440 strain metabolizes glucose through a cycle formed by enzymes of the Entner-Doudoroff, Embden-Meyerhof-Parnas, and pentose phosphate pathways. J. Biol. Chem. 290, 25920–25932. 10.1074/jbc.M115.687749 26350459 PMC4646247

[B25] Radkov A. D. Moe L. A. (2013). Amino acid racemization in *Pseudomonas putida* KT2440. J. Bacteriol. 195, 5016–5024. 10.1128/JB.00761-13 23995642 PMC3811597

[B26] Radkov A. D. Moe L. A. (2014a). Amino acid racemase enzyme assays. Bio Protoc. 4:e1112 10.21769/BioProtoc.1112

[B27] Radkov A. D. Moe L. A. (2014b). Bacterial synthesis of d-amino acids. Appl. Microbiol. Biotechnol. 98, 5363–5374. 10.1007/s00253-014-5726-3 24752840

[B28] Revelles O. Espinosa-urgel M. Fuhrer T. Sauer U. Ramos J. L. (2005). Multiple and interconnected pathways for l-lysine catabolism in *Pseudomonas putida* KT2440. J. Bacteriol. 187, 7500–7510. 10.1128/JB.187.21.7500-7510.2005 16237033 PMC1272968

[B29] Revelles O. Wittich R.-M. Ramos J. L. (2007). Identification of the initial steps in d-lysine catabolism in *Pseudomonas putida*. J. Bacteriol. 189, 2787–2792. 10.1128/JB.01538-06 17259313 PMC1855791

[B30] Studier F. W. (2005). Protein production by auto-induction in high-density shaking cultures. Protein Expr. Purif. 41, 207–234. 10.1016/j.pep.2005.01.016 15915565

[B31] Vollmer W. Blanot D. de Pedro M. A. (2008). Peptidoglycan structure and architecture. FEMS Microbiol. Rev. 32, 149–167. 10.1111/j.1574-6976.2007.00094.x 18194336

[B32] Vranova V. Zahradnickova H. Janous D. Skene K. R. Matharu A. S. Rejsek K. (2012). The significance of d-amino acids in soil, fate and utilization by microbes and plants: review and identification of knowledge gaps. Plant Soil 354, 21–39. 10.1007/s11104-011-1059-5

[B33] Wasserman S. A. Walsh C. T. Botstein D. (1983). Two alanine racemase genes in *Salmonella typhimurium* that differ in structure and function. J. Bacteriol. 153, 1439–1450. 6298185 10.1128/jb.153.3.1439-1450.1983 PMC221795

[B34] Watanabe S. Morimoto D. Fukumori F. Shinomiya H. Nishiwaki H. Kawano-Kawada M.. (2012). Identification and characterization of d-hydroxyproline dehydrogenase and Δ^1^-pyrroline-4-hydroxy-2-carboxylate deaminase involved in novel l-hydroxyproline metabolism of bacteria. J. Biol. Chem. 287, 32674–32688. 10.1074/jbc.M112.374272 22833679 PMC3463351

