# Supplementary material for: A Broad Spectrum Racemase in Pseudomonas putida KT2440 Plays a Key Role in Amino Acid Catabolism
Source: Front Microbiol. 2018 Jun 29;9:1343. doi: 10.3389/fmicb.2018.01343 (PMC6034422; doi:10.3389/fmicb.2018.01343)
Supplement: Figure S1 — OD600 values for growth of wt and Δalr strains on 19 L-AAs. [file Data_Sheet_1.docx]

Figure S1

Figure S2

Table S1

| Protein fraction | Percent Alkaline phosphatase activity ^a^  (Established periplasm localization) | | Percent Glucose-6-phosphate dehydrogenase activity ^a^  (Established cytoplasm localization) | |
| --- | --- | --- | --- | --- |
|  | Wild-type | Δalr | Wild-type | Δalr |
| Periplasmic | 77.00 ± 9.99 | 72.42 ± 5.19 | 16.72 ± 1.64 | 13.32 ± 4.12 |
| Total | 100 ± 0.00 | 100 ± 0.00 | 100.00 ± 0.00 | 100.00 ± 0.00 |
| ^a^ Enzyme activity in the “Total” fraction was normalized to 100% for the alkaline phosphatase and glucose-6-phosphate dehydrogenase activity assays | | | | |
